# Variability of the Atmospheric PM_10_ Microbiome in Three Climatic Regions of France

**DOI:** 10.3389/fmicb.2020.576750

**Published:** 2021-01-13

**Authors:** Abdoulaye Samaké, Jean M. F. Martins, Aurélie Bonin, Gaëlle Uzu, Pierre Taberlet, Sébastien Conil, Olivier Favez, Alexandre Thomasson, Benjamin Chazeau, Nicolas Marchand, Jean-Luc Jaffrezo

**Affiliations:** ^1^University Grenoble Alpes, CNRS, IRD, INP-G, IGE (UMR 5001), Grenoble, France; ^2^University Grenoble Alpes, CNRS, LECA (UMR 5553), BP 53, Grenoble, France; ^3^ANDRA DRD/OPE Observatoire Pérenne de l’Environnement, Bure, France; ^4^INERIS, Parc Technologique Alata, BP 2, Verneuil-en-Halatte, France; ^5^AtmoAuvergne-Rhônes Alpes, Grenoble, France; ^6^Aix Marseille Univ, CNRS, LCE, Marseille, France

**Keywords:** airborne microorganisms, bioaerosol, regional sources, bacteria, fungi, sugar compounds, DNA metabarcoding, climatic gradient

## Abstract

Primary Biogenic Organic Aerosols (PBOA) were recently shown to be produced by only a few types of microorganisms, emitted by the surrounding vegetation in the case of a regionally homogeneous field site. This study presents the first comprehensive description of the structure and main sources of airborne microbial communities associated with temporal trends in Sugar Compounds (SC) concentrations of PM_10_ in 3 sites under a climatic gradient in France. By combining sugar chemistry and DNA Metabarcoding approaches, we intended to identify PM_10_-associated microbial communities and their main sources at three sampling-sites in France, under different climates, during the summer of 2018. This study accounted also for the interannual variability in summer airborne microbial community structure (bacteria and fungi only) associated with PM_10_-SC concentrations during a 2 consecutive years’ survey at one site. Our results showed that temporal changes in PM_10_-SC in the three sites are associated with the abundance of only a few specific taxa of airborne fungi and bacterial. These taxa differ significantly between the 3 climatic regions studied. The microbial communities structure associated with SC concentrations of PM_10_ during a consecutive 2-year study remained stable in the rural area. Atmospheric concentration levels of PM_10_-SC species varied significantly between the 3 study sites, but with no clear difference according to site typology (rural vs. urban), suggesting that SC emissions are related to regional rather than local climatic characteristics. The overall microbial beta diversity in PM_10_ samples is significantly different from that of the main vegetation around the urban sites studied. This indicates that the airborne microorganisms at these urban sites are not solely from the immediate surrounding vegetation, which contrasts with observations at the scale of a regionally homogeneous rural site in 2017. These results improve our understanding of the spatial behavior of tracers of PBOA emission sources, which need to be better characterized to further implement this important mass fraction of Organic Matter (OM) in Chemical Transport models (CTM).

## Introduction

Primary biogenic organic aerosols (PBOAs) are basically a subgroup of atmospheric organic particles that are directly introduced from the biosphere to the atmosphere (Després et al., 2012). PBOAs include many types of biological particles, notably including living and non-living organisms (e.g., bacteria, viruses, green algae, microbial fragments, etc.), dispersal units (e.g., plant pollen, fungal and bacterial spores, etc.) and other types of biological materials (e.g., plant and pollen debris, etc.) (Forde et al., 2019; Huffman et al., 2020). They constitute a major fraction of the total concentration of organic matter (OM) in the atmosphere (Coz et al., 2010; Bozzetti et al., 2016). For instance, it has recently been shown that the contribution of PBOA to the mass load of organic aerosols in PM_10_ is comparable to that of secondary aerosol collected at a rural background site in Switzerland in both winter and summer (Bozzetti et al., 2016). PBOAs are subject of increasing research interest, not least because of the growing evidence of their adverse effects on human health and agricultural issues (e.g., allergic asthma, aspergillosis, rhinitis, damage to food crops, etc.) and their influence on the hydrological cycle and climate by acting as condensation or ice nuclei in mixed-phase clouds (Després et al., 2012; Forde et al., 2019). These various impacts are likely to be effective on a regional scale due to the transport of PBOA-containing air masses (Després et al., 2012; Yu et al., 2016).

Although recent studies have revealed useful information on the size, segregation, and abundance of specific PBOA components, quantitative studies on the overall contribution to OM and their predominant atmospheric emission processes are still relatively scarce (Bozzetti et al., 2016; Yu et al., 2016). As a result, the estimation of global emissions of terrestrial PBOAs into the atmosphere is still relatively poorly constrained, ranging from 50 to 1,000 Tg y^–1^ (Boucher et al., 2005; Jaenicke, 2005; Yu et al., 2016), indicating that considerable uncertainties in the modeling of their physico-chemical influences in a climate system still remain (Bozzetti et al., 2016; Yu et al., 2016). Consequently, the need for more studies to better quantify the atmospheric loading of PBOAs on regional and global scales has been highlighted in recent studies (Yu et al., 2016; Helin et al., 2017). Such information would certainly allow better parameterization of source-resolved chemical transport models (CTM), which are still generally unable to accurately simulate the relevant fractions of OM (Heald et al., 2011; Ciarelli et al., 2016).

Primary sugar compounds (SC, defined here as sugar alcohols and primary saccharides), emitted continuously from biological sources, are one of the main water-soluble organic compounds present in atmospheric aerosols (Medeiros and Simoneit, 2007; Li et al., 2018; Yan et al., 2019). Due to their ubiquity and abundance, specific SCs have been used as relevant markers to describe sources and estimate the contributions of PBOAs to the OM mass in the atmosphere (Zhu et al., 2015; Kang et al., 2018; Samaké et al., 2019a). For example, glucose and trehalose (also called mycose) are the most common primary saccharides in vascular plants and are an important source of carbon for soil microorganisms (e.g., bacteria, fungi, etc.) (Medeiros and Simoneit, 2007; Pietrogrande et al., 2014). Although other sources of glucose in atmospheric aerosols have been suggested in a few previous studies, such as biomass combustion and marine emissions (Yang et al., 2012; Zhu et al., 2015), these two chemical compounds have been used mainly as generic markers for soil biota emitted to the atmosphere from natural soil suspension and agricultural soils (Medeiros and Simoneit, 2007; Pietrogrande et al., 2014). Similarly, sugar alcohols, particularly mannitol and arabitol, have long been proposed as markers for airborne fungi, and have been used to estimate their contribution to the mass of PBOAs in various studies around the world (Bauer et al., 2008; Golly et al., 2018; Li et al., 2018). Sugar alcohols, especially arabitol and mannitol are together an important fraction of the dry weight of fungi as they are both intracellular osmoprotectant and common storage carbohydrates (Medeiros and Simoneit, 2007; Pietrogrande et al., 2014).

Our recent study on daily (24 h) filter samples of particulate matter with aerodynamic diameter below 10 μm (PM_10_) collected simultaneously at 16 sites, grouped by sets of increasing spatial scales in France, revealed very synchronous temporal trends in SC concentrations and species ratio at city scale and scales up to 200 km (Samaké et al., 2019b). Such a pattern indicates that the processes responsible for the evolution of SC concentrations in PM_10_ show spatial homogeneity over typical areas of at least tens of kilometers, which could most probably be attributed to a very dynamic assemblage in the airborne microbiome that is strongly influenced by the local environment (e.g., meteorological conditions, land use, etc.) (Forde et al., 2019). Nevertheless, little is known about the identity of airborne microbial communities (i.e., community composition and diversity) associated with the temporal dynamics of atmospheric SC concentrations. In this context, our recent taxonomic analyses conducted in summer 2017 at a rural agricultural site in France showed that the daily dynamics in PM_10_ SC concentrations are clearly determined by the abundance of only a few specific taxa of airborne fungi and bacteria measured at that study site (Samaké et al., 2020). As a follow-up, the present study aimed to use the DNA metabarcoding approach (Taberlet et al., 2012) to identify PM_10_-associated microbial communities and their main sources at three climatically very different sampling sites in France during the summer of 2018. Our main objectives were 2-fold: (i) to examine the interannual variability of the structure of summer airborne microbial community (bacteria and fungi only) associated with PM_10_ SC concentrations during a consecutive 2-year survey, (ii) to determine whether the airborne microbial community structure associated with PM_10_ SC concentrations differs between 3 different French climatic conditions in order to test the hypothesis of local rather than regional emissions of PBOA and associate SC. The results of this study can provide insight into the structure of airborne microbiomes reflecting the spatiotemporal dynamics of PM_10_ SC concentrations, which is essential to improve the modeling accuracy of PBOA emission processes in CTM models.

## Materials and Methods

### Site Description and Sampling Strategy

**FIGURE 1:**
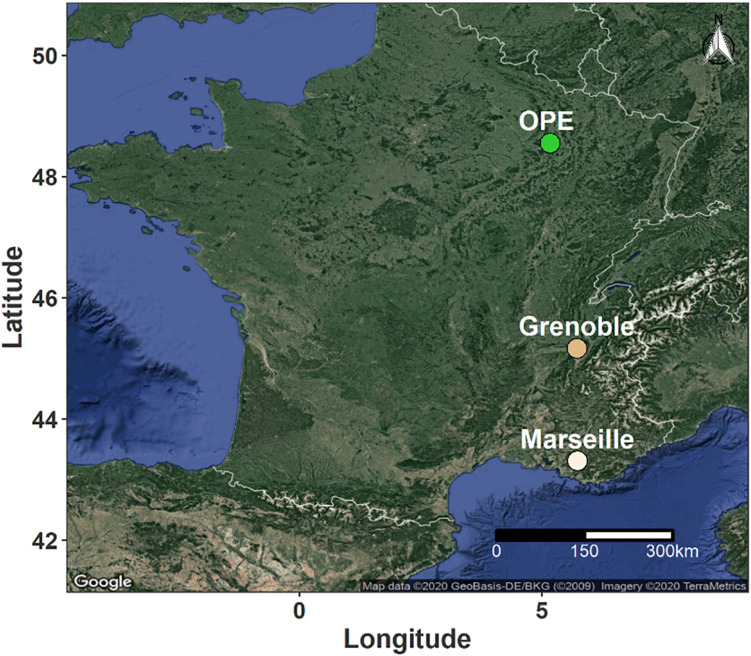
Geographical location of the sampling sites in France. The green dot indicates a rural background site in an area of intensive agriculture, while the light and dark beige dots correspond to urban background sites in Mediterranean and Alpine environments, respectively.

Samples of PM_10_ aerosols were collected at three representative sites in different geographic regions of France. The sampling sites were selected to cover several main types of environmental conditions in terms of site topography, climate, land use and cover (Figure 1). Two urban background sites (Grenoble and Marseille) and one rural background site (OPE) were selected for this study. These sites are respectively located in alpine (Grenoble, 45°09’41” N, 5°44’07” E, 220 m a.s.l.), mediterranean (Marseille, 43°18’20” N, 5°23’40” E, 64 m a.s.l.) and continental (OPE, 48°56’2” N; 5°5’ E, 392 m a.s.l.) areas of France. The detailed geographical characteristics of the sampling sites have been described previously (Favez et al., 2010; Salameh et al., 2015; Samaké et al., 2020) and will only be briefly described here. In short, the city of Grenoble is by far the most densely populated urban area in the French Alps and is located at the junction of three mountain ranges (Favez et al., 2010). Marseille is the second most populated city in France and displays a large port area that extends over almost 70 km of the Mediterranean coastline (Salameh et al., 2015). The two sampling sites are part of French regional air quality monitoring networks. Finally, the OPE is a specific long-term multi-disciplinary observatory, with no major sources of pollution nearby (Samaké et al., 2020). The sampling site itself is located in the middle of an intensive agricultural area and is surrounded by field crops (several tens of kilometers in all directions) (Samaké et al., 2020).

Two seasonal aerosol sampling campaigns were conducted during the summers of 2017 and 2018 at the study sites. PM_10_ aerosol samples were collected according to the methodology described previously (Samaké et al., 2019b) using a high-volume sampler (DA80, Digitel; 24 h at 30 m^3^ h^–1^). In brief, PM_10_ samples were collected onto quartz fiber filters (Tissue-quartz PALL QAT-UP 2500 150 mm diameter) pre-heated 6 h at 500°C to remove potential trace of organic contaminants. One 150 mm filter is obtained over a 24 h cycle (starting at 9 am UTC to 9 am UTC the next day) and no replicate is done according to a national procedure validated for atmospheric aerosols sampling: a filtration at 30 m^3^/h for 24 h produces a homogenous deposition on the surface area within 5%. Then the filter is kept to freezer prior analysis. More details can be found in the recommendations: (EN 16450:2017^1^; EN 12341:2014^2^). All the analyses are done on the same filter after punching some parts directed toward different analysis or extraction before their analysis. The summer 2018 monitoring campaign was achieved simultaneously at the three sites and samples were taken at different frequencies: 1 day out of 6 for the OPE site from 14/05 to 30/08 (16 samples), every 3 days for Grenoble from 23/06 to 31/08 (24 samples) and on a daily basis for the Marseille sites from 05/07 to 25/07 (20 samples).

At the OPE site, a daily collection of filters has already been carried out during the summer 2017 (June to August) (Samaké et al., 2020), allowing to assess the inter-annual variation in the microbial composition of PM_10_. Field blank filters (about 10% of the samples) were obtained by setting the filters in the sampler without air flow during the sampling period. They were handled as real samples for quality assurance purposes. DNA extraction and PCR controls were also included. The dataset presented here has been corrected from negative controls.

A previous source-tracking analysis indicated that airborne microbial communities are most likely the result of high intakes from nearby sources, whereas intakes from distant sources are low and diluted (Tignat-Perrier et al., 2019). Since our previous study showed that the local vegetation was the main sources of airborne microbial communities in relation to SC concentrations at the OPE site (Samaké et al., 2020), leaf samples of the main vegetation type within 100 meters of each aerosol sampler were collected at the same time as the PM_10_ sampling. After collection, the samples were stored in airtight containers (sterile bottles, Schott, GL45, 100 ml) containing 15 g of silica gel. More details on sampling procedures and storage conditions are reported elsewhere (Samaké et al., 2020). Approximately 10 to 15 vegetation samples were analyzed per site, which is probably sufficient to conclude about local influence but is clearly insufficient to have an exhaustive image of the microbial communities from regions beyond the immediate surroundings of the urban sites. Due to unfortunate circumstances, leaf samples collected at the OPE site were lost during transport to the laboratory. This is not, however, a prohibitive factor for this study as we had already been able to observe stable SC concentrations trends over 7 consecutive years at this site.

### Chemical Analyses

Details on the chemical analysis of aerosol are presented elsewhere (Golly et al., 2018; Samaké et al., 2019a,b). Briefly, each aerosol sample was analyzed for various chemical species using subsampled fractions of the collection filters and a wide range of analytical methods. In particular, SCs (i.e., arabitol, mannitol, trehalose, and glucose) were systematically analyzed in PM_10_ samples by high-performance liquid chromatography with pulsed amperometric detection (Waked et al., 2014; Golly et al., 2018). Elemental and organic carbon (EC, OC) were analyzed using a Sunset Lab. instrument and following the EUSSAR2 thermo-optical protocol (Cavalli et al., 2010).

### Biological Analyses

#### DNA Extraction

Aerosol samples pre-treatment and DNA extraction experiments were performed according to an optimized protocol, as presented elsewhere (Samaké et al., 2020). Briefly, 2 filter punches of a diameter of 38 mm each were extracted with the DNeasy PowerWater kit (QIAGEN, Germantown, MD) according to the supplier’s instructions, with the following modifications (Samaké et al., 2020): Elution of biological materials on the polyethersulphone membrane disc filter (PES, Supor^®^ 47 mm 200, 0.2 μm, PALL), 30 min sonication at 65°C in a thermal water bath (EMAG, Emmi-60 HC, Germany; 50% of efficiency), 5 min bead-beating before and after sonication, and the DNA was finally eluted with 50 μL EB buffer.

Leave samples were dried in contact of about 30 g of silica gel. To extract DNA from epiphytic or endophytic microorganisms, aliquots of leaf samples (approx. 25–30 mg) were crushed using a Tissue-Lyser (QIAGEN, Germany). DNA was extracted with the DNeasy Plant Mini Kit (QIAGEN, Germany) according to the original manufacturer’s protocol, with some minor modifications as already detailed elsewhere (Samaké et al., 2020).

#### PCR Amplification and Sequencing

The PCR amplification procedures and materials used in this study were similar to those described in our recent work (Samaké et al., 2020). Briefly, the hypervariable V4 region of the bacterial 16S rRNA gene was amplified using the Bact02 primer (Forward 5 Boyer et al., 2016), so that all pairwise tag combinations were differentiated by at least five different base pairs (Taberlet et al., 2018).

Four independent PCR replicates were performed for each DNA extract. Amplification of the bacterial 16S rDNA gene and fungal ITS1 region was performed in a 20 μL PCR reaction mixture containing 10 μL of AmpliTaq Gold 360 Master Mix (Applied Biosystems, Foster City, CA, United States), 0.16 μL of 20 mg ml^–1^ bovine serum albumin (BSA; Roche Diagnostics, Basel, Switzerland), 0.2 μM of each primer, and 2 μL of diluted DNA extract. The DNA extracts from the air samples were diluted eight times, while DNA extracts from the leaves were diluted four times. The thermal cycling program was as follows: an initial activation of DNA polymerase for 10 min at 95°C; 40 denaturation cycles of 30 s at 95°C, 30 s annealing at 53°C and 56°C for Bacteria and Fungi, respectively, 90 s elongation at 72°C; and a final elongation at 72°C for 7 min. Approximately 10% of the PCR products were randomly selected and controlled using a QIAxel Advance device (QIAGEN, Hilden, Germany) equipped with a high-resolution separation cartridge.

After amplification, PCR products from the same marker were pooled in equal volumes and purified with the MinElute kit (Qiagen, Hilden, Germany) following the manufacturer’s instructions. Both pools were sent to Fasteris SA (Geneva, Switzerland) for library preparation and MiSeq Illumina 2 × 250 bp paired-end sequencing. The two sequencing libraries (one per marker) were prepared and libraries as are presented elsewhere (Samaké et al., 2020), which aims to limit the formation of chimeras. Negatives of DNA extraction and of PCR, as well as unused tag combinations were included in the sequencing experiment to monitor for potential false positives due to tag jumps and contamination (Schnell et al., 2015).

#### Bioinformatics Analyses of Raw Sequences

The raw read sequences were processed separately for each library using the OBITools software suite (Boyer et al., 2016) and the detailed workflow has recently been presented elsewhere (Samaké et al., 2020). Note that all raw reads (i.e., 2017 and 2018) have been processed simultaneously. In short, the raw paired-ends were assembled and the low-quality sequences (Fastq average quality score < 40) were rejected. The aligned sequences were then assigned to the corresponding PCR replicates allowing zero and two mismatches on the tags and primers, respectively. Strictly identical sequences were de-replicated and a base filtration step was performed to select sequences within the expected range length (i.e., longer than 65 or 39 bp for fungi and bacteria, respectively, excluding tags and primers), without ambiguous nucleotides, and observed at least 10 times in at least one PCR replicate.

The remaining unique sequences were then clustered into Molecular Taxonomic Units (MOTUs) at a 97% threshold using Sumatra and Sumaclust algortihms (Mercier et al., 2013). Taxonomy assignments of each MOTU were made to a reference set of full-length metabarcodes using the open reference *ecotag* program (Boyer et al., 2016). The reference database was built with the *ecoPCR* program (Ficetola et al., 2010) for each library based on the version 113 of the EMBL database. The resulting datasets were then processed with the open source software R (R studio interface, version 3.4.1) to filter out chimeric sequences, potential contaminants and failed PCR replicates. Finally, the remaining PCR replicates were summed per sample. The final datasets, as well as the OBITools commands applied, have been uploaded to the DRYAD repository (doi: 10.5061/dryad.dv41ns1wf).

#### Statistical Analyses

All statistical analyses were carried out with R (R Core Team, 2017). First of all, raw MOTU abundances were rarefied at the same sequencing depth as before any statistical analyses to cope with the heterogeneity of the number of sequences per sample. The rarefaction and extrapolation curves were obtained with the *iNext 2.0-12* package (Hsieh et al., 2016), to study the gain in species richness as we increased the sequencing depth for each sample. The alpha diversity estimator was calculated using the *phyloseq* package *1.22-3* (McMurdie and Holmes, 2013) to estimate the Chao1 index and Shannon diversity. The similarity of microbial communities was represented by non-metric multidimensional scale (NMDS) using the Morisita-Horn similarity distance metric. The NMDS ordination analyses were performed with the *metaMDS* function within the *vegan* package (Oksanen et al., 2020) with the number of random starts set to 999. The samples were also visualized using hierarchical cluster analysis based on the same dissimilarity index. An analysis of similarities (ANOSIM) was performed to assess whether sample categories contained significantly different microbial communities. An analysis of homogeneity of variance was performed with the *betadisper* function in the *vegan* package to test whether airborne microbial compositions differed in their dispersion over time. The null hypothesis was that the mean dispersion within a group was the same across all groups. The relationship between airborne microbial communities and PM_10_ SC species was assessed using Kendall’s rank correlation.

## Results and Discussion

### Richness and Diversity Indexes of Microbial Communities

The structures of airborne bacterial and fungal communities (the fraction of micro-eucaryotes was not considered here) were obtained for the 121 samples collected, consisting of 96 aerosols and 25 leaf samples. After paired-end assembly of reads, sample assignment, and stringent quality filtering (including chimera and contaminant filtering), 4,678,172 and 8,721,309 sequences were obtained in total for the bacterial and fungal dataset, respectively. These sequences represented 3,460 bacterial and 1,490 fungal MOTUs in all collected samples. The rarefaction curves of MOTU richness revealed common logarithmic shapes approaching a plateau for each sample (Supplementary Figure S1), indicating that most bacterial and fungal species were detected in our samples. Alpha-diversity estimators, including Chao1 and Shannon indices, were calculated to estimate the mean richness and biodiversity of microbial communities in PM_10_ samples analyzed at each study site. MOTU tables were refined to 2,468 and 8,072 bacterial and fungal sequence reads respectively, prior to diversity analyses. As shown in Supplementary Figure S2, the microbial diversity and richness of PM_10_ differed significantly at each sampling site (*p* < 0.05), confirming that the diversity and composition of atmospheric bacteria and fungi in aerosol samples are most likely determined by the surrounding landscape and their corresponding local climatic conditions. On average, the Chao1 richness estimators and Shannon diversity indices of the bacterial MOTU in Marseille were the highest (*p* < 0.05), while that of the OPE site was the lowest (*p* < 0.05). Chao1 values of airborne fungi were highest in Grenoble and lowest at the OPE site (*p* < 0.05), while the Shannon index values remained similar at the different study sites.

We also compared the microbial diversity of PM_10_ during two consecutive summer periods at the rural OPE site. Samples from summer 2017 had the highest richness (number of MOTUs) in bacterial and fungal MOTUs (*p* < 0.05), while, the summer 2018 samples presented approximately the highest bacterial and fungal MOTU diversity (Richness and Evenness).

### Microbial Community in Airborne PM_10_

#### Airborne Bacterial Community Composition

**FIGURE 2:**
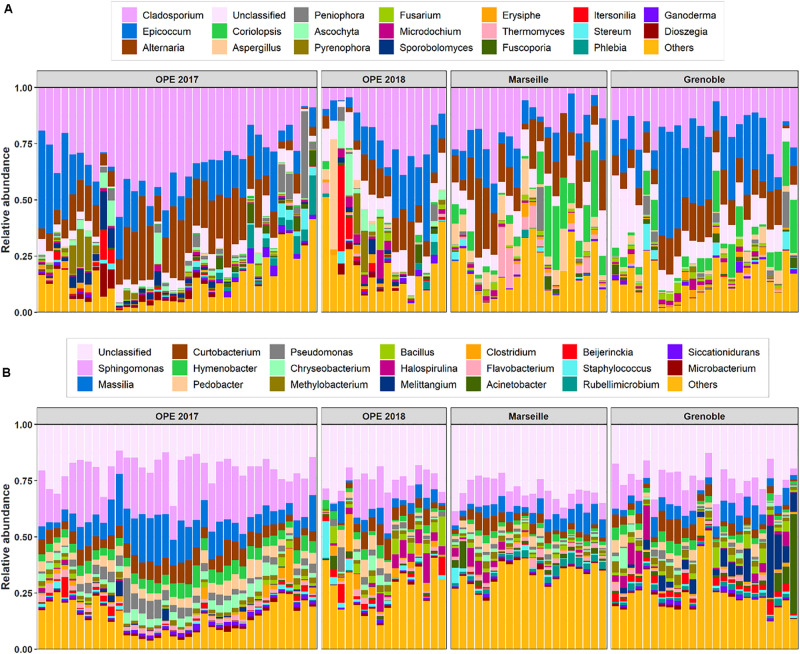
Relative abundance of the top 20 fungi **(A)** and bacteria **(B)** detected in PM_10_ samples at all sites. The samples are grouped by site location. X-axis corresponds to each individual daily sample ranged in chronological order.

In total, the airborne bacterial microbiome was divided into 24 microbial phyla, 54 classes, 124 orders, and 275 families for all PM_10_ samples. Despite very different geographical conditions, the average bacterial sequence reads (mean ± *SD*) were dominated in the three sites by Proteobacteria (48.7 ± 10.7%), followed by Bacteroidetes (18.9 ± 6.1%), Actinobacteria (16.5 ± 5.1%), Firmicutes (9.2 ± 7.4%), Cyanobacteria (1.9 ± 3.2%), with less than 3% of the total bacterial sequence reads unclassified. These phyla have been commonly observed in the atmosphere (Maron et al., 2005; Liu et al., 2019; Wei et al., 2020). Proteobacteria constitute a major taxonomic group among prokaryotes, and have previously been reported as the most abundant bacterial phylum in air samples from central northern France (Maron et al., 2005). At the class level, the predominant Bacteria are Alphaproteobacteria (25.5 ± 6.4%), Actinobacteria (15.8 ± 4.9%), Gammaproteobacteria (10.6 ± 7.3%), Betaproteobacteria (8.0 ± 4.9%), Cytophagia (6.4 ± 2.5%), Bacilli (6.2 ± 4.6%), Sphingobacteriia (6.0 ± 3.0%), Flavobacteriia (4.0 ± 2.3%), Clostridia (2.5 ± 3.6%), and Deltaproteobacteria (2.3 ± 4.7%). Up to 650 genera were detected in all aerosol samples, although many sequences (24.5 ± 6.7%) could not be taxonomically attributed at the genus level. The most abundant genera (>2%, see Figure 2B) are *Sphingomonas* (13.9 ± 7.3%), followed by *Massilia* (7.4 ± 5.0%), *Curtobacterium* (5.0 ± 2.6%), *Hymenobacter* (3.3 ± 1.9%), *Pedobacter* (3.1 ± 2.0%), *Pseudomonas* (2.3 ± 2.4%), *Chryseobacterium* (2.1 ± 1.8%), and *Methylobacterium* (1.9 ± 1.0%).

#### Airborne Fungal Community Composition

In all aerosol samples, fungal communities were characterized by 3 phyla, 24 classes, 73 orders, and 212 families. The fungal sequence reads in air samples of the three sites are consistently dominated by the Ascomycota phylum (76.5 ± 19.1%), followed by the Basidiomycota (23.4 ± 19.0%), which are known to actively eject spores into the atmosphere along with aqueous jets or droplets containing a mixture of inorganic solutes and carbohydrates into the atmosphere (Després et al., 2007). The remaining sequences are affiliated to Mucoromycota (<0.03%) and to unclassified sequences (approximately 0.03%). This is consistent with the results of previous studies also indicating that the subkingdom of Dikarya (Ascomycota and Basidiomycota) accounts for 98% of known species in the biological kingdom of Eumycota (i.e., fungi) in the atmosphere (James et al., 2006; Fröhlich-Nowoisky et al., 2009). Among these observed phylotypes, the predominant classes (>1%) are Dothideomycetes (69.4 ± 19.9%), followed by Agaricomycetes (18.1 ± 18.9%), Eurotiomycetes (5.7 ± 8.1%), Sordariomycetes (5.5 ± 6.3%), Leotiomycetes (3.7 ± 3.9%), Tremellomycetes (3.2 ± 5.8%), and Microbotryomycetes (1.1 ± 2.3%). The predominant orders are Pleosporales (3.2 ± 5.8%) and Capnodiales (3.2 ± 5.8%), which belong to Ascomycota. Similarly, the dominant orders in Basidiomycota are Polyporales (10.2 ± 10.5%), followed by Russulales (3.2 ± 6.8%) and Tremellales (2.0 ± 2.6%). At the genus level, about 528 taxa are characterized in all air samples (Figure 2A), of which *Cladosporium* (24.0 ± 12.2%), *Epicoccum* (15.7 ± 11.2%), *Alternaria* (13.7 ± 8.0%), *Coriolopsis* (4.1 ± 7.1%), *Aspergillus* (2.5 ± 5.8%), and *Peniophora* (1.9 ± 5.0%) are the most abundant communities.

#### Interannual Variation in the Airborne Microbe Composition at the OPE Site

**FIGURE 3:**
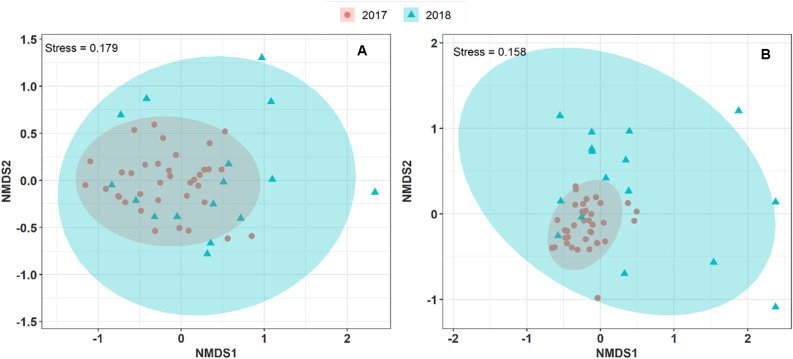
Non-metric multi-dimensional scale ordination plots (NMDS) of airborne microbial community composition over two consecutive summer periods at the OPE site. NDMS plots are constructed from a Horn distance matrix of MOTUs abundances for fungi **(A)** and bacteria **(B)**, respectively. The data sets are rarefied at the same sequencing depth. The stress values indicate an adequate two-dimensional picture of the sample distribution. The ellipses represent 95% confidence intervals for the cluster centroids.

To explore and visualize the variation of airborne microbial communities in PM_10_ collected during two consecutive summer periods at the OPE site, we performed an NMDS ordination analysis based on Morishita-Horn dissimilarity distance matrices. As shown in Figure 3, the airborne bacterial and fungal communities in the aerosol samples collected in summer 2017 and 2018 are closely grouped. An unsupervised hierarchical cluster analysis shows a pattern similar to that observed on the NMDS ordination, where the airborne bacterial and fungal MOTUs in PM_10_ are clustered together regardless of the sampling year. It indicates that the structure of the predominant airborne bacterial and fungal community has remained stable over the years at the OPE site (Supplementary Figure S3). This result is logical since agricultural practices (types of crops, harvests, etc.) have been shown to drive airborne microbial communities (Mhuireach et al., 2016; Wei et al., 2019). Although crops around the OPE observatory are subject to a 3-year rotation system, overall crops in the vicinity do not vary significantly from 1 year to the next (Samaké et al., 2019b). However, the microbial communities observed in summer 2018 are dispersively distributed with longer distances between samples (Betadisper, *p* < 0.05) than in summer 2017, for both fungi and bacteria. This result could be, at least partially, attributed to the lower aerosol sampling frequency applied in 2018.

#### Spatial Variation in the Composition of Airborne Microbes

**FIGURE 4:**
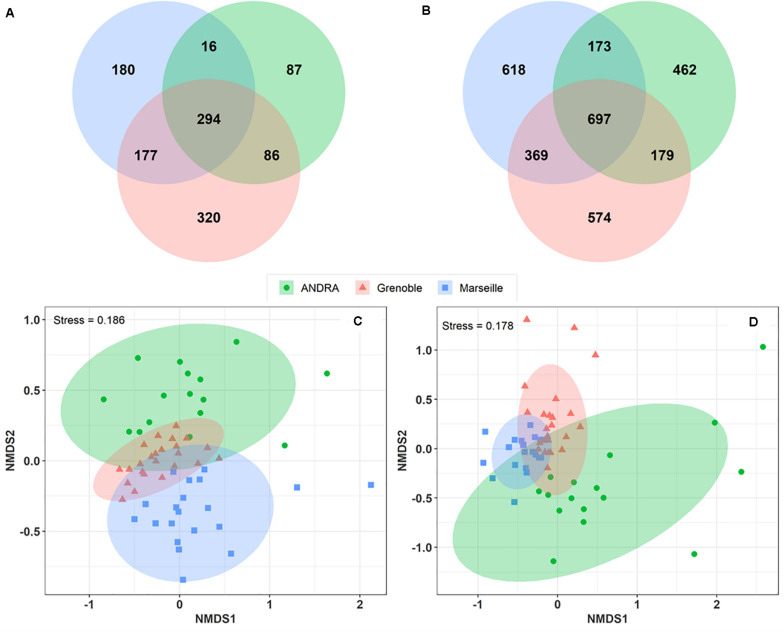
Venn diagrams showing the numbers of fungal **(A)** and bacterial **(B)** MOTUs shared between study sites. Only those MOTUs detected at least 10 times in each sample were used to construct the Venn diagrams. Comparison of the composition of PM_10_ samples by geographical sites on a NMDS scaling ordination **(C,D)**. The NDMS plots are constructed from a Horn distance matrix of MOTUs abundances for fungi **(A)** and bacteria **(B)**, respectively. The data sets are rarefied at the same sequencing depth. The stress values indicate an adequate two-dimensional picture of samples distribution. The ellipses represent 95% confidence intervals for the cluster centroids.

A Venn diagram was constructed to analyze the overlap of MOTUs on the pairs of sites (Figures 4A,B). A total of 177 fungal MOTUs are shared between the urban background sites of Marseille and Grenoble, whereas the rural OPE site shared only 16 and 86 MOTUs respectively with the Marseille and Grenoble sites. Similarly, 369 bacterial MOTUs are shared between the Marseille and Grenoble sites, whereas the bacterial community of OPE shared only 173 and 179 MOTUs with the Marseille and Grenoble site, respectively. Summer PM_10_ samples from the urban sites shared the highest proportion of microbial MOTUs, suggesting that the Marseille and Grenoble sites tend to have more similar airborne bacterial and fungal community compositions. This suggests that agricultural practices have a different influence on the composition of the local microbiome in the air than urbanization activities.

To study the overall similarity and disparity of microbial community between sites, a NMDS ordination analysis based on the Horn distance matrix was performed (Figures 4C,D). Figures 4C,D show that, in general, PM_10_ samples from the same location are closely grouped together. Further similarity analysis (ANOSIM, overall *R* = 0.28 and *R* = 0.34 with *p* < 0.01 for fungal and bacterial communities, respectively) confirmed the significant difference between the 3 distinct clusters by geographic locations. These results indicate that the bacterial and fungal communities in PM_10_ samples are grouped by geographical region, although some taxa are common to all three sites.

#### Spatial and Temporal Variability in Concentrations of Primary Sugar Compounds

A large database on concentrations of PM_10_ SC at study sites has been acquired previously at many sites all over France (Samaké et al., 2019a,b), including the three sites of this study. It has already been shown that arabitol, mannitol, glucose, and trehalose are generally the main SC species in all urban and rural areas investigated in France (Samaké et al., 2019a). Their mean annual and summer concentrations at the 3 sites of the present study for the years 2017 and 2018 are shown in Table 1. This table shows that the concentration of individual SC species at the Grenoble site remained almost constant throughout the 2 years of sampling, indicating a reproducibility in the concentration trends of SC species. At the OPE site, mean concentrations of individual SC species in 2018 are generally lower than in 2017. However, it should be noted that this result may have been biased by the difference in sampling frequency used in summer 2018 (1 every 6 days) compared to 2017 (daily). This is important because the source contribution is episodic in nature (e.g., harvesting) (Samaké et al., 2020), therefore the most intense episodes were not necessarily collected.

**TABLE 1:** Abundances (mean ± standard deviation) of four major primary sugar compounds measured at each study site over two campaigns (2017 and 2018).

	**OPE**		**Grenoble**		**Marseille**	
**Compounds**	**Annual**	**Summer**	**Annual**	**Summer**	**Annual**	**Summer**
**2017**	**(*n* = 107)**	**(*n* = 64)**	**(*n* = 123)**	**(*n* = 31)**	**(*n* = 66)**	**(*n* = 15)**
Arabitol	46.2 ± 71.8	69.4 ± 85.0	19.6 ± 21.2	39.1 ± 27.3	8.0 ± 7.8	12.0 ± 6.8
Mannitol	46.0 ± 65.9	69.2 ± 76.4	21.5 ± 22.7	43.9 ± 22,1	6,9 ± 6,1	11.6 ± 6.7
Glucose	36.1 ± 33.8	48.3 ± 37.5	26.8 22.0	44.5 ± 25,6	11.7 ± 7.4	15.8 ± 8.1
Trehalose	34.6 ± 47.5	53.7 ± 53.1	10.9 ± 15.4	24.0 ± 19.8	NA	NA

**2018**	**(*n* = 57)**	**(*n* = 16)**	**(*n* = 186)**	**(*n* = 71)**		**(*n* = 21)**

Arabitol	16.4 ± 19.4	37.9 ± 25.3	24.8 ± 23,4	38.5 ± 25.1	NA	151.2 ± 81.5
Mannitol	20.8 ± 20.8	37.3 ± 24.0	25.8 ± 22,0	40.5 ± 19.2	NA	164.6 ± 67.4
Glucose	25.6 ± 30.3	36.3 ± 37,8	33.1 ± 27,9	37.7 ± 19.7	NA	155.8 ± 128.7
Trehalose	4.5 ± 10.3	8.4 ± 15.5	9.2 ± 14.6	12.9 ± 15.7	NA	85.9 ± 43.7

*In 2018, data for the Marseille site were only available in July. NA denotes not measured. Summer period corresponds to June–July–August.*

As also shown in Table 1, the ambient concentration of the main SC species varies significantly between the sites, but with no clear difference according to the site typology (rural vs. urban). This suggests that SC emissions are less site specific but more related with regional climatic characteristics.

#### Relationship Between Primary Sugar Compounds and Dominant Airborne Microbial MOTUs

**FIGURE 5:**
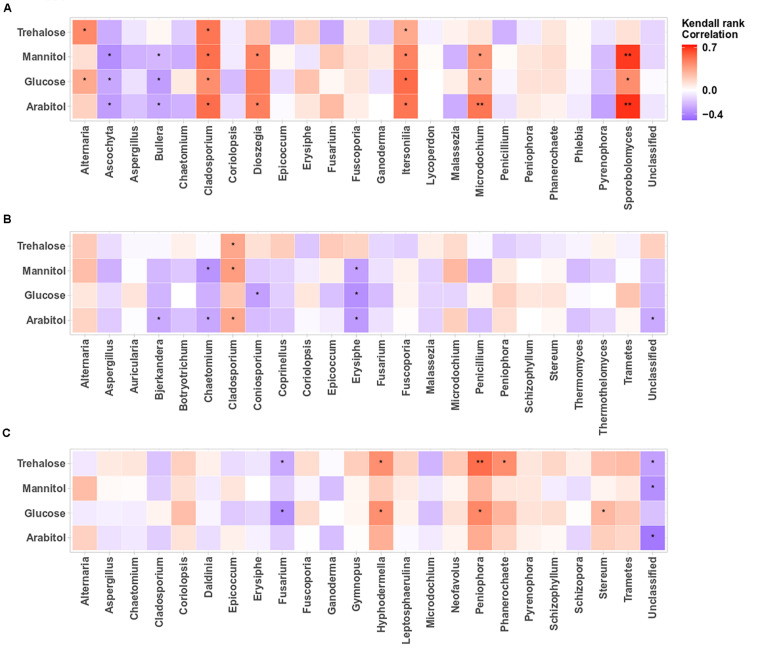
Heatmaps of Kendall’s rank correlation between SCs and abundance of airborne fungal communities at the study sites: **(A–C)** correspond to the OPE, Marseille and Grenoble sites, respectively. Only the 24 most abundant fungal genera (relative abundance ≥1) are indicated. Asterisks indicate significant correlations (**0.001 < *p* < 0.01, *0.01 < *p* < 0.05).

**FIGURE 6:**
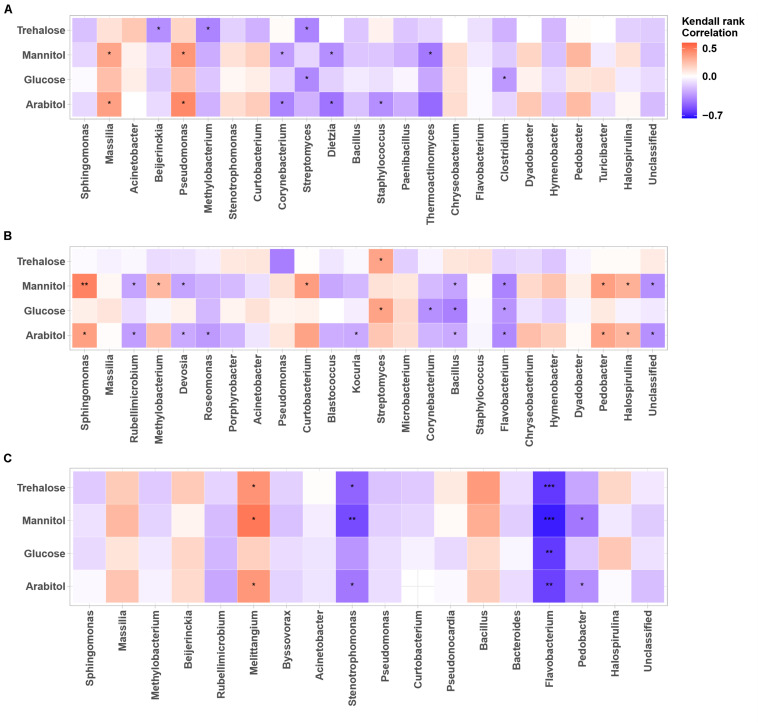
Heatmaps of Kendall’s rank correlation between SCs and abundance of airborne bacterial communities at the study sites: **(A–C)** correspond to the OPE, Marseille and Grenoble sites, respectively. Only the 24 most abundant fungal genera (relative abundance ≥1) are indicated. Asterisks indicate significant correlations (^∗∗∗^0.00001 < *p* < 0.001, ^∗∗^0.001 < *p* < 0.01, ^∗^0.01 < *p* < 0.05).

The structure of airborne PM_10_ microbial taxa associated with the temporal trends in atmospheric SC concentration levels may vary in different geographical areas. Therefore, we calculated the Kendall’s rank correlation between the dominant SC species (i.e., arabitol, mannitol, glucose, trehalose) and the abundance of airborne microbial community at genus level. This analysis reveals variable relationships among sampling sites (Figures 5A–C, 6A–C). At the OPE site, the temporal dynamics of SC species is mainly better correlated (*p* < 0.05) with the fungal genera *Alternaria, Cladosporium*, *Dioszegia, Itersonilia, Microdochium*, and *Sporobolomyces* (Figure 5A). This observation is consistent with that made in summer 2017 at the same site (Samaké et al., 2020). Similarly, at the Marseille site (Figure 5B), time series of SC species are positively correlated (*p* < 0.05) only with *Cladosporium*, and to a lesser degree (not significant) with the fungal genus *Alternaria*, whereas they are best correlated (*p* < 0.05) with the abundances of fungal genera *Hyphodermella, Phanerochaete, Peniophora*, and *Stereum* at the Grenoble site (Figure 5C).

As for airborne fungi, we also found that the temporal dynamics of concentration levels of SC species are positively correlated with the abundances of the bacterial genera *Massilia* (*p* < 0.05) and *Pseudomonas* (*p* < 0.05) at the OPE site (Figure 6A), whereas it is better correlated (*p* < 0.05) with the abundances of the bacterial genera *Sphingomonas, Curtobacterium, Streptomyces, Pedobacter, and Halospirulina* at the Marseille site (Figure 6A). Some bacterial genera, notably *Melittangium*, also showed a strong positive correlation (*p* < 0.05) with PM_10_ SC species at the Grenoble site (Figure 6C). These results clearly show site-specific microbiome signatures measured in the PM_10_ of the 3 sites, with overlaps of only few fungal and bacterial genera. Overall, these observed regional trends probably result from ecosystems heterogeneity, such as site topography, microclimate, vegetation structure, around our study sites. A recent study has shown that the atmospheric microbiome composition is indeed primarily structured by the surrounding landscape types (Tignat-Perrier et al., 2019).

#### Potential Sources of Airborne Microbial Communities at Study Sites

**FIGURE 7:**
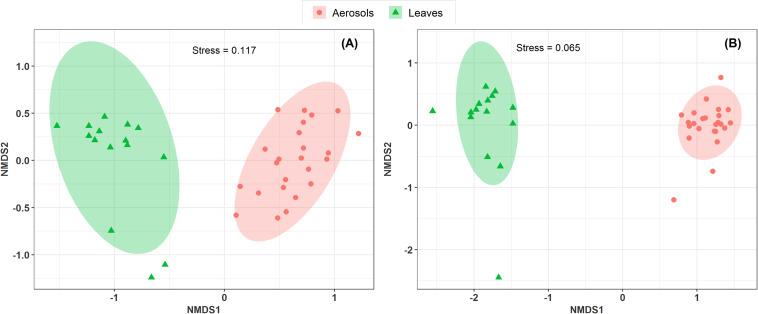
Comparison of the composition of sample types in a NMDS scaling ordination. The NDMS plots are constructed from a Horn distance matrix of MOTUs abundances for fungi **(A)** and bacteria **(B)**, respectively. The data sets are rarefied at the same sequencing depth. The stress values indicate an adequate two-dimensional picture of the sample distributions. The ellipses represent 95% confidence intervals for the cluster centroids. The circular and triangular symbols highlight respectively PM_10_ in air and in leaf samples from the Grenoble site.

**FIGURE 8:**
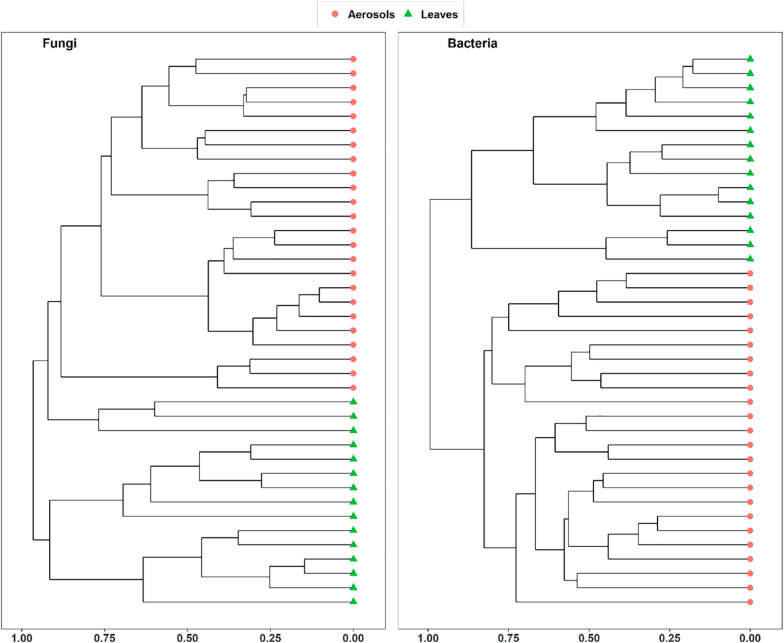
Unsupervised hierarchical clustering of aerosol and leaf samples collected at Grenoble site during summer 2018. Dissimilarity matrixes, based on Horn distance matrix, have been calculated on the rarefied MOTUs tables.

Vegetation structure has been proposed as one of the main sources of airborne microorganisms in urban background areas (Genitsaris et al., 2017). A recent study has shown that the episodic daily fluctuations in PM_10_ SC concentrations at the Grenoble site are very often well synchronized with those of cellulose (Samaké et al., 2019b), a suitable molecular tracer of atmospheric plant biomass (Puxbaum and Tenze-Kunit, 2003). The latter study in Grenoble (Samaké et al., 2019b) also reported maximum atmospheric concentrations of SCs when the vegetation density indicator (leaf area index) was at its maximum in late summer. Therefore, to determine whether the atmospheric microbial genera most positively correlated with SC species were derived from surrounding vegetation, the overall microbial beta diversity in leaf samples of the main vegetation around the two urban stations was analyzed. For the Grenoble site, the NMDS ordination analysis (Figure 7) shows that the overall beta diversities are very similar within the same habitat (PM_10_ or plant) and are very different from a habitat to another (ANOSIM, *R* = 0.89 and 0.97, *p* < 0.01 for fungal and bacterial communities, respectively). These results are also confirmed by an unsupervised hierarchical cluster analysis, which reveals a pattern similar to that observed in the NMDS ordinate (Figure 8), where airborne PM_10_ taxa are grouped separately from those in leaf samples. Similar results were also obtained for the Marseille site (Supplementary Figure S4). These results suggest that the airborne microorganisms at the urban sites studied here do not originate only from the immediate surrounding vegetation. To validate this suggestion, specific samplings of PM_10_ and vegetation should be conducted beyond all immediate surroundings of the urban sites, although this appears a complicate, if not impossible, task, being the large number of potential and heterogeneous sources. In this study, the collection of vegetation samples was clearly not exhaustive and microorganisms typical of each site may have been missed, and do not appear in our analysis. Although not fully supported by direct evidence, these results clearly invalidate, however, our initial hypothesis of specific local emissions of PBOA and SC at urban sites. In other words, airborne microbes in urban areas are probably of allochthonous origin, as already suggested in other contexts (Genitsaris et al., 2017; Tignat-Perrier et al., 2019), with influence on emissions within a radius of several tens of kilometers (Tignat-Perrier et al., 2019).

## Conclusion

This study presents the first comprehensive description of the structure and main sources of airborne microbial communities associated with temporal trends in PM_10_ SC concentration levels at 3 French sites with different climatic conditions. The following main conclusions could be drawn from the results obtained:

• Temporal trends of SC in PM_10_ in the three sites are associated with the abundance of only a few specific taxa of airborne fungal and bacterial. These microbial taxa differ significantly between the 3 different climatic zones studied.
• The structure of summer airborne microbial community associated with PM_10_ SC concentrations during a consecutive 2 year survey remains stable at one site in an agricultural area.
• Atmospheric concentration levels of PM_10_ SC species vary significantly between the 3 study sites, but without clear differences according to site typology (rural vs urban), suggesting that SC emissions are not only local, around the site, but also related to regional climatic characteristics.
• The overall microbial beta diversity in PM_10_ samples are significantly different from that of the main vegetation around the urban sites studied. This indicates that the airborne microorganisms at the urban sites studied here do not originate only from the immediate surrounding vegetation, and are likely to be of allochthonous origin, although no direct evidence could be provided, given the great difficulty in comprehensively sampling the wide variety of potential sources at long distances. The hypothesis of only specific local emissions in urban areas is however ruled out as already suggested in recent studies. This is a different conclusion to that obtained for our rural site in 2017 with homogeneous agricultural practices on a large scale.

Overall, these results improve our understanding of the spatial behavior of tracers of PBOA emission sources in general, which is imperative for further implementation of this large OM mass fraction into CTM models. However, contributions from remote sources in urban areas still need to be validated through specific sampling. Similarly, direct validation of the important SC contents of some species among the dominant microbial taxa identified in each site of this study still needs to be performed, after growth under controlled laboratory conditions. This will allow to decipher the environmental conditions controlling the profile and concentration levels of the SC species produced by the dominant microorganisms, relevant in PBOA emissions.

## Data Availability Statement

The datasets presented in this study can be found in online repositories. The names of the repository/repositories and accession number(s) can be found below: 10.5061/dryad.dv41ns1wf.

## Author Contributions

GU, J-LJ, and JM supervised the thesis of AS. GU and J-LJ designed the atmospheric chemistry part of the Mobil’Air program. AT and SC supervised the sampling at Grenoble and ANDRA sites, respectively. BC and NM supervised the sampling at Marseille site. AS, AB, and JM designed the microbiological aspects of the study. AS and AB performed the experiments. AB performed the bioinformatic analyses. AS performed statistical analyses and wrote the original manuscript. All authors revised the manuscript.

## Conflict of Interest

The authors declare that the research was conducted in the absence of any commercial or financial relationships that could be construed as a potential conflict of interest.

## Footnotes

1 Ambient Air—Automated Measuring Systems for the Measurement of The Concentration of Particulate Matter (PM_10_; PM_2_._5_); Technical Report EN 16450:2017; CEN: Paris, France, 2017.

2 Ambient Air—Standard Gravimetric Measurement Method for the Determination of the PM_10_ or PM_2_._5_ Mass Concentration of Suspended Particulate Matter; Technical Report EN 12341:2014; CEN: Paris, France, 2014.

## References

[B1] Bauer H. Claeys M. Vermeylen R. Schueller E. Weinke G. Berger A. (2008). Arabitol and mannitol as tracers for the quantification of airborne fungal spores. *Atmos. Environ.* 42 588–593. 10.1016/j.atmosenv.2007.10.013

[B2] Boucher O. Randall D. Artaxo P. Bretherton C. Feingold G. Forster P. (2005). “Clouds and Aerosols,” in *Climate Change 2013: The Physical Science Basis. Contribution of Working Group I to the Fifth Assessment Report of the Intergovernmental Panel on Climate Change*, eds Qin D. Plattner G.-K. Tignor M. Allen S. K. Boschung J. Nauels A. (Cambridge: Cambridge University Press), 571–892.

[B3] Boyer F. Mercier C. Bonin A. Le Bras Y. Taberlet P. Coissac E. (2016). OBITOOLS: a UNIX -inspired software package for DNA metabarcoding. *Mol. Ecol. Resour.* 16 176–182. 10.1111/1755-0998.12428 25959493

[B4] Bozzetti C. Daellenbach K. R. Hueglin C. Fermo P. Sciare J. Kasper-Giebl A. (2016). Size-resolved identification, characterization, and quantification of primary biological organic aerosol at a european rural site. *Environ. Sci. Technol.* 50 3425–3434. 10.1021/acs.est.5b05960 26900965

[B5] Cavalli F. Viana M. Yttri K. E. Genberg J. Putaud J.-P. (2010). Toward a standardised thermal-optical protocol for measuring atmospheric organic and elemental carbon: the EUSAAR protocol. *Atmos. Meas. Tech.* 3 79–89. 10.5194/amt-3-79-2010

[B6] Ciarelli G. Aksoyoglu S. Crippa M. Jimenez J.-L. Nemitz E. Sellegri K. (2016). Evaluation of European air quality modelled by CAMx including the volatilitybasis set scheme. *Atmos. Chem. Phys.* 16 10313–10332. 10.5194/acp-16-10313-2016

[B7] Coz E. Artíñano B. Clark L. M. Hernandez M. Robinson A. L. Casuccio G. S. (2010). Characterization of fine primary biogenic organic aerosol in an urban area in the northeastern United States. *Atmos. Environ.* 44 3952–3962. 10.1016/j.atmosenv.2010.07.007

[B8] Després V. R. Alex Huffman J. Burrows S. M. Hoose C. Safatov A. S. Buryak G. (2012). Primary biological aerosol particles in the atmosphere: a review. *Tellus B* 64:15598.

[B9] Després V. R. Nowoisky J. F. Klose M. Conrad R. Andreae M. O. Pöschl U. (2007). Characterization of primary biogenic aerosol particles in urban, rural, and high-alpine air by DNA sequence and restriction fragment analysis of ribosomal RNA genes. *Biogeosciences* 4 1127–1141. 10.5194/bg-4-1127-2007

[B10] Favez O. Haddad I. E. Piot C. Boreave A. Abidi E. Marchand N. (2010). Inter-comparison of source apportionment models for the estimation of wood burning aerosols during wintertime in an Alpine city (Grenoble, France). *Atmos. Chem. Phys.* 10 5295–5314. 10.5194/acp-10-5295-2010

[B11] Ficetola G. Coissac E. Zundel S. Riaz T. Shehzad W. Bessière J. (2010). An In silico approach for the evaluation of DNA barcodes. *BMC Genom.* 11:434. 10.1186/1471-2164-11-434 20637073 PMC3091633

[B12] Forde E. Gallagher M. Foot V. Sarda-Esteve R. Crawford I. Kaye P. (2019). Characterisation and source identification of biofluorescent aerosol emissions over winter and summer periods in the United Kingdom. *Atmos. Chem. Phys.* 19 1665–1684. 10.5194/acp-19-1665-2019

[B13] Fröhlich-Nowoisky J. Pickersgill D. A. Després V. R. Pöschl U. (2009). High diversity of fungi in air particulate matter. *Proc. Natl. Acad. Sci. U.S.A.* 106 12814–12819. 10.1073/pnas.0811003106 19617562 PMC2722276

[B14] Genitsaris S. Stefanidou N. Katsiapi M. Kormas K. A. Sommer U. Moustaka-Gouni M. (2017). Variability of airborne bacteria in an urban Mediterranean area (Thessaloniki, Greece). *Atmos. Environ.* 157 101–110. 10.1016/j.atmosenv.2017.03.018

[B15] Golly B. Waked A. Weber S. Samake A. Jacob V. Conil S. (2018). Organic markers and OC source apportionment for seasonal variations of PM2.5 at 5 rural sites in France. *Atmos. Environ.* 198 142–157. 10.1016/j.atmosenv.2018.10.027

[B16] Heald C. L. Coe H. Jimenez J. L. Weber R. J. Bahreini R. Middlebrook A. M. (2011). Exploring the vertical profile of atmospheric organic aerosol: comparing 17 aircraft field campaigns with a global model. *Atmos. Chem. Phys.* 11 12673–12696. 10.5194/acp-11-12673-2011

[B17] Helin A. Sietiö O.-M. Heinonsalo J. Bäck J. Riekkola M.-L. Parshintsev J. (2017). Characterization of free amino acids, bacteria and fungi in size-segregated atmospheric aerosols in boreal forest: seasonal patterns, abundances and size distributions. *Atmos. Chem. Phys.* 17 13089–13101. 10.5194/acp-17-13089-2017

[B18] Hsieh T. C. Ma K. H. Chao A. (2016). iNEXT: an R package for rarefaction and extrapolation of species diversity (Hill numbers). *Methods Ecol. Evol.* 7 1451–1456. 10.1111/2041-210X.12613

[B19] Huffman J. A. Perring A. E. Savage N. J. Clot B. Crouzy B. Tummon F. (2020). Real-time sensing of bioaerosols: review and current perspectives. *Aerosol Sci. Technol.* 54 465–495. 10.1080/02786826.2019.1664724

[B20] Jaenicke R. (2005). Abundance of cellular material and proteins in the atmosphere. *Science* 308 73–73. 10.1126/science.1106335 15802596

[B21] James T. Y. Kauff F. Schoch C. L. Matheny P. B. Hofstetter V. Cox C. J. (2006). Reconstructing the early evolution of Fungi using a six-gene phylogeny. *Nature* 443 818–822. 10.1038/nature05110 17051209

[B22] Kang M. Ren L. Ren H. Zhao Y. Kawamura K. Zhang H. (2018). Primary biogenic and anthropogenic sources of organic aerosols in Beijing, China: insights from saccharides and n-alkanes. *Environ. Pollut.* 243 1579–1587. 10.1016/j.envpol.2018.09.118 30293040

[B23] Li L. Ren L. Ren H. Yue S. Xie Q. Zhao W. (2018). Molecular characterization and seasonal variation in primary and secondary organic aerosols in Beijing, China. *J. Geophys. Res. Atmos.* 123 12394–12412. 10.1029/2018JD028527

[B24] Liu H. Hu Z. Zhou M. Hu J. Yao X. Zhang H. (2019). The distribution variance of airborne microorganisms in urban and rural environments. *Environ. Pollut.* 247 898–906. 10.1016/j.envpol.2019.01.090 30823344

[B25] Maron P.-A. Lejon D. P. H. Carvalho E. Bizet K. Lemanceau P. Ranjard L. (2005). Assessing genetic structure and diversity of airborne bacterial communities by DNA fingerprinting and 16S rDNA clone library. *Atmos. Environ.* 39 3687–3695. 10.1016/j.atmosenv.2005.03.002

[B26] McMurdie P. J. Holmes S. (2013). Phyloseq: an R package for reproducible interactive analysis and graphics of microbiome census data. *PLoS One* 8:e61217. 10.1371/journal.pone.0061217 23630581 PMC3632530

[B27] Medeiros P. M. Simoneit B. R. T. (2007). Analysis of sugars in environmental samples by gas chromatography–mass spectrometry. *J. Chromatogr. A* 1141 271–278. 10.1016/j.chroma.2006.12.017 17207493

[B28] Mercier C. Boyer F. Kopylova E. Taberlet P. Bonin A. Coissac E. (2013). SUMATRA and SUMACLUST: fast and exact comparison and clustering of sequences. *Programs Abstr. SeqBio Workshop* 14 27–28.

[B29] Mhuireach G. Johnson B. R. Altrichter A. E. Ladau J. Meadow J. F. Pollard K. S. (2016). Urban greenness influences airborne bacterial community composition. *Sci. Total Environ.* 571 680–687. 10.1016/j.scitotenv.2016.07.037 27418518

[B30] Oksanen J. Blanchet F. G. Friendly M. Kindt R. Legendre P. McGlinn D. (2020). *Vegan: Community Ecology Package.* Available online at: https://cran.r-project.org/web/packages/vegan/index.html (accessed February 10, 2020).

[B31] Pietrogrande M. C. Bacco D. Visentin M. Ferrari S. Casali P. (2014). Polar organic marker compounds in atmospheric aerosol in the Po Valley during the Supersito campaigns — Part 2: seasonal variations of sugars. *Atmos. Environ.* 97 215–225. 10.1016/j.atmosenv.2014.07.056

[B32] Puxbaum H. Tenze-Kunit M. (2003). Size distribution and seasonal variation of atmospheric cellulose. *Atmos. Environ.* 37 3693–3699. 10.1016/s1352-2310(03)00451-5

[B33] R Core Team (2017). *R: A Language and Environment for Statistical Computing*. Available online at: https://www.R-project.org/

[B34] Salameh D. Detournay A. Pey J. Pérez N. Liguori F. Saraga D. (2015). PM2.5 chemical composition in five European Mediterranean cities: a 1-year study. *Atmos. Res.* 155 102–117. 10.1016/j.atmosres.2014.12.001

[B35] Samaké A. Bonin A. Jaffrezo J.-L. Taberlet P. Weber S. Uzu G. (2020). High levels of primary biogenic organic aerosols are driven by only a few plant-associated microbial taxa. *Atmos. Chem. Phys.* 20 5609–5628. 10.5194/acp-20-5609-2020

[B36] Samaké A. Jaffrezo J.-L. Favez O. Weber S. Jacob V. Albinet A. (2019a). Polyols and glucose particulate species as tracers of primary biogenic organic aerosols at 28 French sites. *Atmos. Chem. Phys.* 19 3357–3374. 10.5194/acp-19-3357-2019

[B37] Samaké A. Jaffrezo J.-L. Favez O. Weber S. Jacob V. Canete T. (2019b). Arabitol, mannitol, and glucose as tracers of primary biogenic organic aerosol: the influence of environmental factors on ambient air concentrations and spatial distribution over France. *Atmos. Chem. Phys.* 19 11013–11030. 10.5194/acp-19-11013-2019

[B38] Schnell I. B. Bohmann K. Gilbert M. T. P. (2015). Tag jumps illuminated - reducing sequence-to-sample misidentifications in metabarcoding studies. *Mol. Ecol. Resour.* 15 1289–1303. 10.1111/1755-0998.12402 25740652

[B39] Taberlet P. Bonin A. Zinger L. Coissac E. (2018). *Environmental DNA: For Biodiversity Research and Monitoring.* Oxford: Oxford University Press.

[B40] Taberlet P. Coissac E. Pompanon F. Brochmann C. Willerslev E. (2012). Towards next-generation biodiversity assessment using DNA metabarcoding: next-generation dna metabarcoding. *Mol. Ecol.* 21 2045–2050. 10.1111/j.1365-294X.2012.05470.x 22486824

[B41] Tignat-Perrier R. Dommergue A. Thollot A. Keuschnig C. Magand O. Vogel T. M. (2019). Global airborne microbial communities controlled by surrounding landscapes and wind conditions. *Sci. Rep.* 9:14441. 10.1038/s41598-019-51073-4 31595018 PMC6783533

[B42] Waked A. Favez O. Alleman L. Y. Piot C. Petit J.-E. Delaunay T. (2014). Source apportionment of PM10 in a north-western Europe regional urban background site (Lens, France) using positive matrix factorization and including primary biogenic emissions. *Atmos. Chem. Phys.* 14 3325–3346. 10.5194/acp-14-3325-2014

[B43] Wei M. Liu H. Chen J. Xu C. Li J. Xu P. (2020). Effects of aerosol pollution on PM2.5-associated bacteria in typical inland and coastal cities of northern China during the winter heating season. *Environ. Pollut.* 262:114188 10.1016/j.envpol.2020.114188 32126435

[B44] Wei M. Xu C. Xu X. Zhu C. Li J. Lv G. (2019). Characteristics of atmospheric bacterial and fungal communities in PM2.5 following biomass burning disturbance in a rural area of North China Plain. *Sci. Total Environ.* 651 2727–2739. 10.1016/j.scitotenv.2018.09.399 30463127

[B45] Yan C. Sullivan A. P. Cheng Y. Zheng M. Zhang Y. Zhu T. (2019). Characterization of saccharides and associated usage in determining biogenic and biomass burning aerosols in atmospheric fine particulate matter in the North China Plain. *Sci. Total Environ.* 650 2939–2950. 10.1016/j.scitotenv.2018.09.325 30373070

[B46] Yang Y. Chan C. Tao J. Lin M. Engling G. Zhang Z. (2012). Observation of elevated fungal tracers due to biomass burning in the Sichuan Basin at Chengdu City, China. *Sci. Total Environ.* 431 68–77. 10.1016/j.scitotenv.2012.05.033 22664540

[B47] Yu X. Wang Z. Zhang M. Kuhn U. Xie Z. Cheng Y. (2016). Ambient measurement of fluorescent aerosol particles with a WIBS in the Yangtze River Delta of China: potential impacts of combustion-related aerosol particles. *Atmos. Chem. Phys.* 16 11337–11348. 10.5194/acp-16-11337-2016

[B48] Zhu C. Kawamura K. Kunwar B. (2015). Organic tracers of primary biological aerosol particles at subtropical Okinawa Island in the western North Pacific Rim: organic biomarkers in the north pacific. *J. Geophys. Res. Atmos.* 120 5504–5523. 10.1002/2015jd023611

