# Supplementary material for: Variability of the Atmospheric PM10 Microbiome in Three Climatic Regions of France
Source: Front Microbiol. 2021 Jan 13;11:576750. doi: 10.3389/fmicb.2020.576750 (PMC7838387; doi:10.3389/fmicb.2020.576750)
Supplement: Supplementary file 1 [file Data_Sheet_1.docx]

**Variability of the atmospheric PM_10_ microbiome in several climatic regions of France**

Abdoulaye Samaké^1^, Jean M.F. Martins^1^*, Aurélie Bonin^2^, Gaëlle Uzu^1^, Pierre Taberlet^2^, Sébastien Conil^3^, Olivier Favez^4^, Alexandre Thomasson^5^, Benjamin Chazeau^6^, Nicolas Marchand^6^, and Jean-Luc Jaffrezo^1^.

^1^University Grenoble Alpes, CNRS, IRD, INP-G, IGE (UMR 5001), 38000 Grenoble, France

^2^University Grenoble Alpes, CNRS, LECA (UMR 5553), BP 53, 38041 Grenoble, France

^3^ANDRA DRD/OPE Observatoire Pérenne de l’Environnement, 55290 Bure, France

^4^INERIS, Parc Technologique Alata, BP 2, 60550 Verneuil-en-Halatte, France

^5^AtmoAuvergne-Rhônes Alpes, 38400 Grenoble, France

^6^Aix Marseille Univ, CNRS, LCE, Marseille, France

** Corresponding author :* Jean Martins ([jean.martins@univ-grenoble-alpes.fr](mailto:jean.martins@univ-grenoble-alpes.fr))


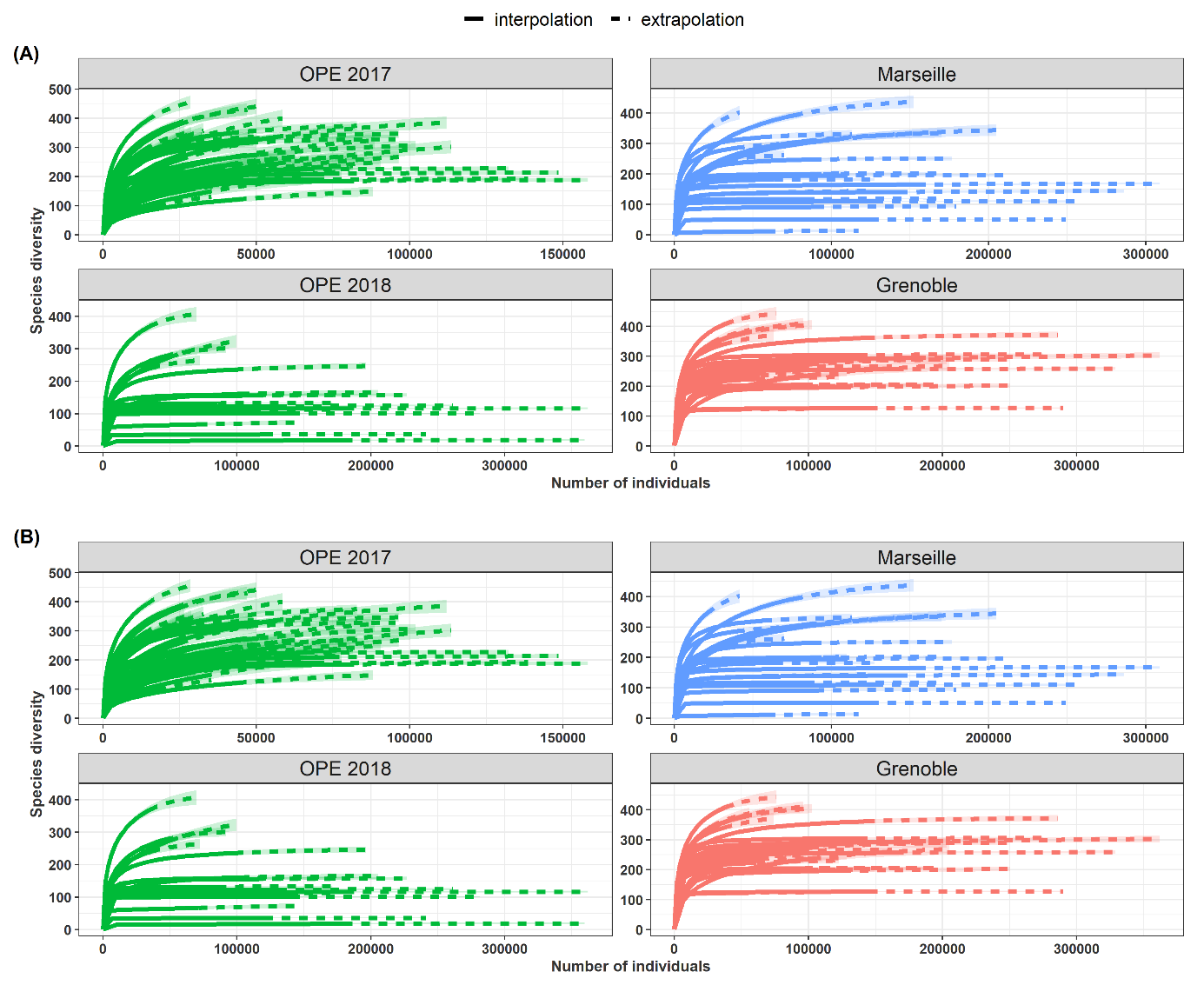


Figure 1 : Rarefaction curves of the airborne microbial MOTUs abundance grouped by sampling sites. (A) Fungal and (B) bacterial MOTUs. Solid curves represent the observations while the dashed ones show the interpolation.


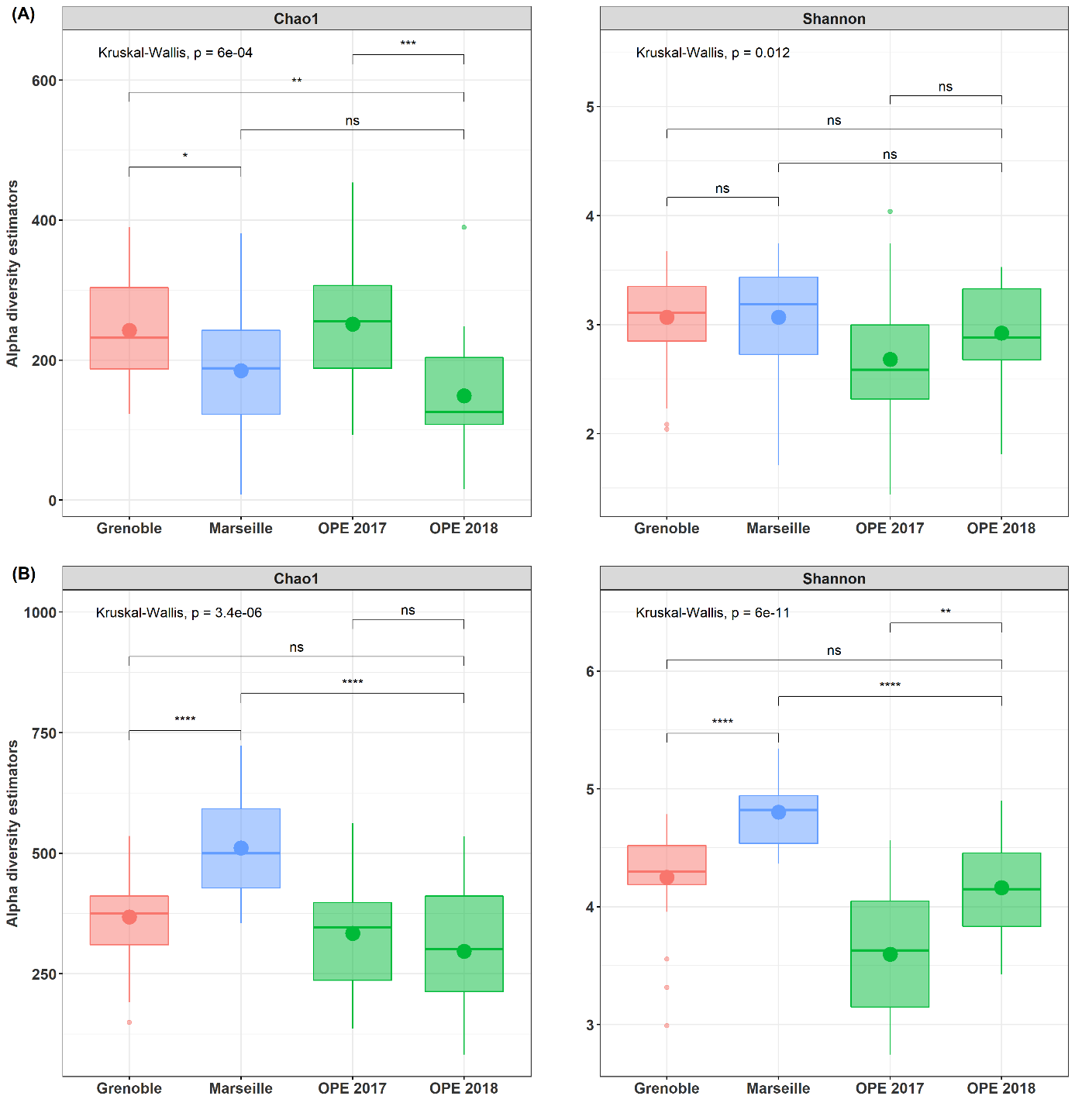


Figure 2 : Statistical comparisons of the richness and diversity of airborne microbial MOTUs between the different sampling sites. Abundance of Fungal (A) and bacterial (B) MOTUs. The symbol inside each box indicates the mean abundance value, while the top, middle, and bottom lines of the box represent the 75th, median, and 25th percentiles, respectively. The whiskers at the top and bottom of the box range from the 95th to the 5th percentile. The data were rarefied at the same minimum sequencing depth. Statistical significance is set at p < 0.05.


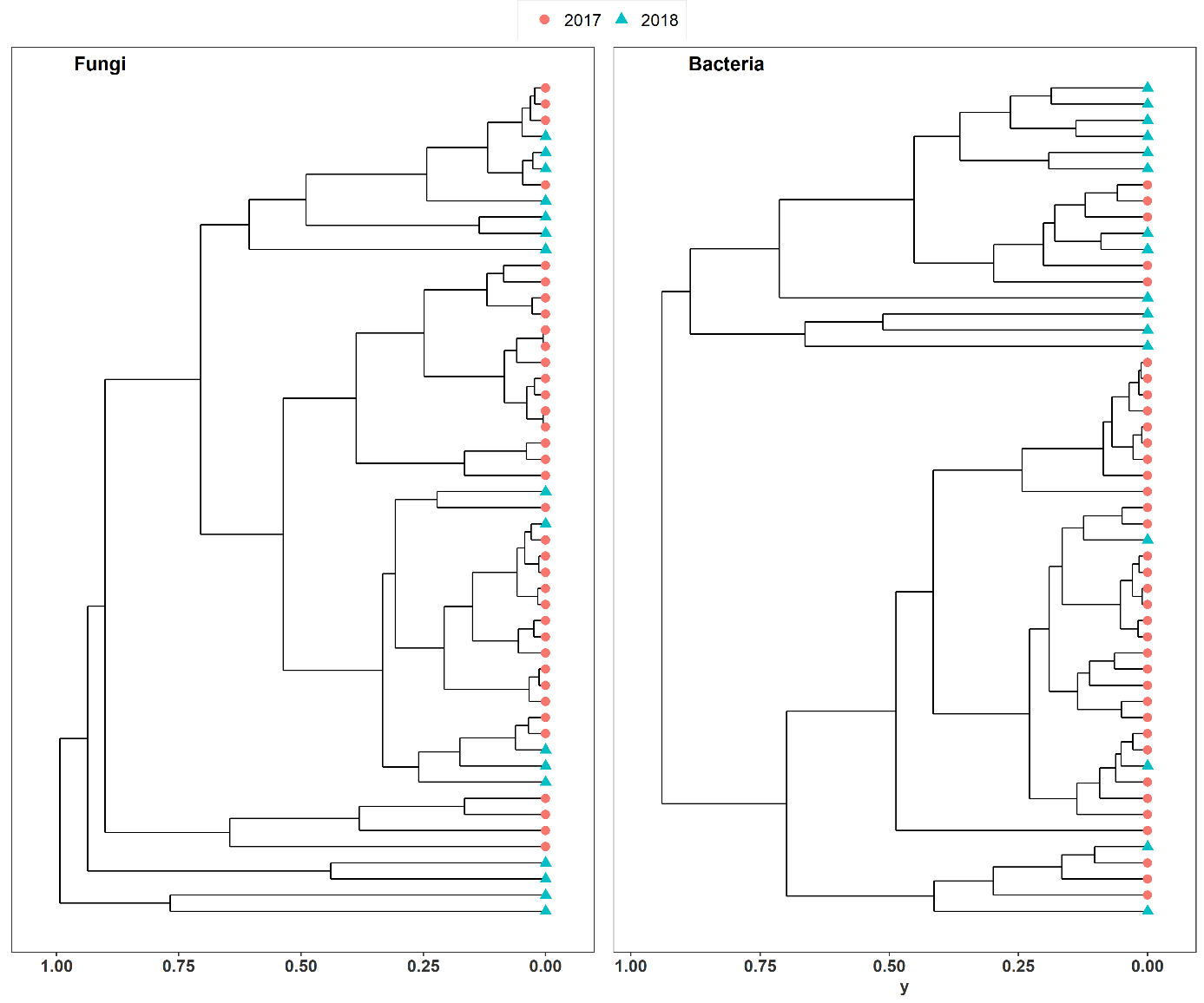


Figure 3 : Unsupervised hierarchical clustering of aerosol samples collected at the OPE site during summer 2017 and 2018. Dissimilarity matrixes, based on Horn distance matrix, have been calculated on the rarefied MOTUs tables.


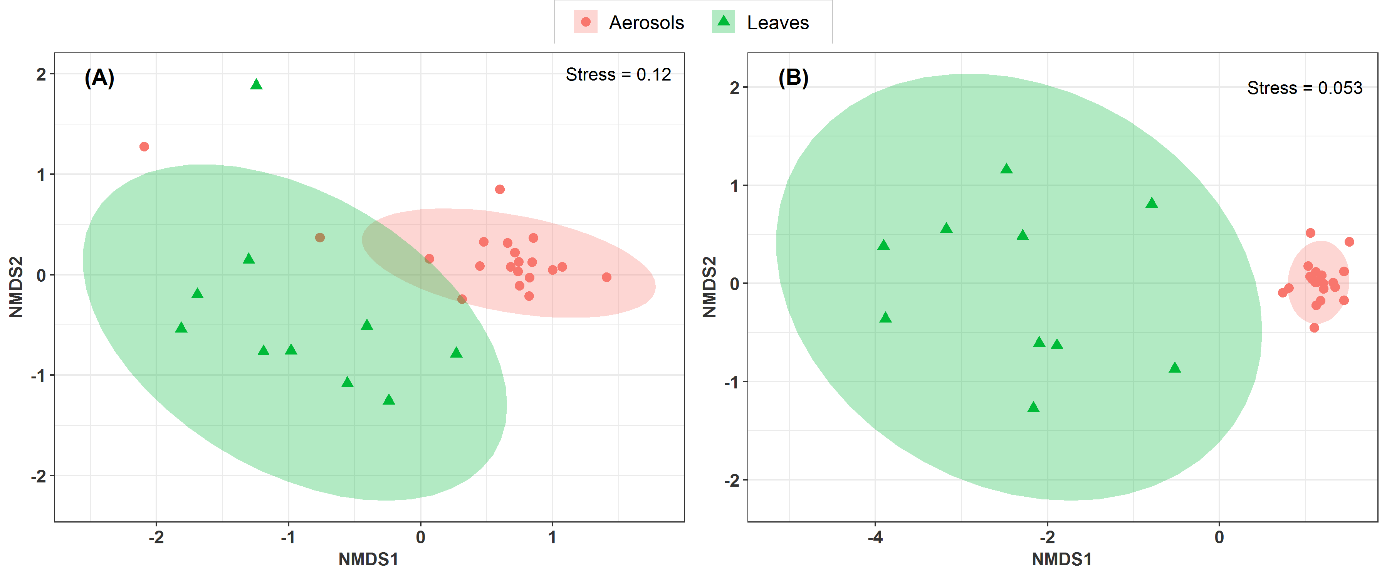


Figure 4 : Compositional comparison of sample types in a NMDS scaling ordination. NDMS plots are constructed from a Horn distance matrix of MOTUs abundances for fungi (A) and bacteria (B), respectively. The data sets were rarefied at the same sequencing depth. The stress values indicate an adequate two-dimensional picture of the samples distribution. The ellipses represent 95% confidence intervals for the cluster centroids. The circular and triangular symbols highlight the air PM_10_ and leaf samples from Marseille site, respectively.
